# Identification of circRNA–miRNA–mRNA Regulatory Network and Crucial Signaling Pathway Axis Involved in Tetralogy of Fallot

**DOI:** 10.3389/fgene.2022.917454

**Published:** 2022-07-07

**Authors:** Zunqi Kan, Wenli Yan, Ning Wang, Yuqing Fang, Huanyu Gao, Yongmei Song

**Affiliations:** ^1^ College of Chinese Medicine, Shandong University of Traditional Chinese Medicine, Jinan, China; ^2^ Institute for Literature and Culture of Chinese Medicine, Shandong University of Traditional Chinese Medicine, Jinan, China

**Keywords:** congenital heart disease, competing endogenous RNA, circular RNA, regulatory networks, tetralogy of fallot

## Abstract

Tetralogy of Fallot (TOF) is one of the most common cyanotic congenital heart diseases (CHD) worldwide; however, its pathogenesis remains unclear. Recent studies have shown that circular RNAs (circRNAs) act as “sponges” for microRNAs (miRNAs) to compete for endogenous RNA (ceRNA) and play important roles in regulating gene transcription and biological processes. However, the mechanism of ceRNA in TOF remains unclear. To explore the crucial regulatory connections and pathways of TOF, we obtained the human TOF gene, miRNA, and circRNA expression profiling datasets from the Gene Expression Omnibus (GEO) database. After data pretreatment, differentially expressed mRNAs (DEmRNAs), microRNAs (DEmiRNAs), and circRNAs (DEcircRNAs) were identified between the TOF and healthy groups, and a global triple ceRNA regulatory network, including circRNAs, miRNAs, and mRNAs based on the integrated data, was constructed. A functional enrichment analysis was performed on the Metascape website to explore the biological functions of the selected genes. Then, we constructed a protein-protein interaction (PPI) network and identified seven hub genes using the cytoHubba and MCODE plug-ins in the Cytoscape software, including BCL2L11, PIK3R1, SOCS3, OSMR, STAT3, RUNX3, and IL6R. Additionally, a circRNA–miRNA–hub gene subnetwork was established, and its enrichment analysis results indicated that the extrinsic apoptotic signaling pathway, JAK-STAT signaling pathway and PI3K-Akt signaling pathway may be involved in the pathogenesis of TOF. We further identified the hsa_circ_000601/hsa-miR-148a/BCL2L11 axis as a crucial signaling pathway axis from the subnetwork. This study provides a novel regulatory network for the pathogenesis of TOF, revealing the possible molecular mechanisms and crucial regulatory pathways that may provide new strategies for candidate diagnostic biomarkers or potential therapeutic targets for TOF.

## 1 Introduction

Tetralogy of Fallot (TOF) is one of the most common congenital heart diseases (CHD), with an estimated incidence of1 in 3500 people or 0.23 to 0.63 cases per 1000 births (Forman et al., 2019). TOF originates from the uneven separation of bulbar and trunk arteries during embryonic development, resulting in malformations such as ventricular septal defects, obstruction of the right ventricular outflow tract, right ventricular hypertrophy, and override of the ventricular septum by the aortic root (Wilson et al., 2019). At present, prenatal diagnosis of TOF mainly relies on fetal echocardiography. The prenatal detection rate of TOF is between 23% and 85.7% (DeVore et al., 2021). Although some indicators can indicate heart malformation in early pregnancy, the diagnosis still needs to be made in the second trimester (Ren et al., 2019; Minnella et al., 2020). Although the advancement of surgical correction in recent years could help repair the structural abnormalities in most patients, it is likely that they will suffer from a series of symptoms, such as heart failure and arrhythmias, which cause considerable burden in their lives (Egbe et al., 2014).

Due to some deficiencies in prenatal diagnosis and surgical treatment of TOF, a growing number of researchers have focused on identifying the mechanisms of TOF (Simmons and Brueckner, 2017; Athanasiadis et al., 2019). The pathogenesis of TOF involves a combination result of genetic, epigenetic, and environmental factors (Bittel et al., 2014). Currently, most studies on the pathogenesis of TOF have focued on mutations in some specific protein-coding genes (GATA4, NKX-2.5, JAG1, FOXC2, TBX5, and TBX1) (Morgenthau and Frishman, 2018), microdeletions of chromosome 22Q11.2, and copy number variations (e.g., 1p21.1) (Mercer-Rosa et al., 2015). However, there is no consensus regarding the exact molecular pathogenesis of TOF. A growing body of evidence supports the role of non-coding RNA (ncRNA) in cardiac disorders, including studies on the role of microRNAs (miRNAs) and messenger RNA (mRNAs) in CHD (Gao J. et al., 2017; Huang et al., 2019). Competing endogenous RNA (ceRNA) refers to the competition between long non-coding RNAs (lncRNAs), circular RNAs (circRNAs), mRNAs, and pseudogenes within the same pool of miRNAs (Tay et al., 2014). Studies have begun to explore the expression and regulatory role of ncRNAs in TOF, such as the expression of small nucleolar RNAs, miRNAs (O'Brien et al., 2012) and lncRNAs (Wang et al., 2018), and the regulatory network of ceRNA related to lncRNAs (Zhang et al., 2021) or circRNAs (Yu et al., 2021). CircRNAs are a particular type of ncRNA molecule that act as miRNA “sponges” to compete for endogenous RNA and participate in the regulation of gene transcription and biological processes. Because of their abundant conserved miRNA reaction elements (MREs) and greater stability than linear RNAs, they have become a new hotspot in research on the ceRNA family (Zhong et al., 2018). Despite the advantages of circRNAs in exploring novel clinical diagnostic biomarkers and therapeutic targets (Kristensen et al., 2019), few studies have reported their role in TOF.

This study aimed to construct a circRNA–miRNA–mRNA and protein–protein interaction (PPI) regulatory network by combining related circRNAs, miRNAs, and mRNAs from the Gene Expression Omnibus (GEO) database (Clough and Barrett, 2016) using bioinformatics methods. It is possible that our findings will help to understand the pathogenesis of TOF and provide early diagnostic markers and potential therapeutic targets.

## 2 Manuscript Formatting

### 2.1 Materials and Methods

**FIGURE 1:**
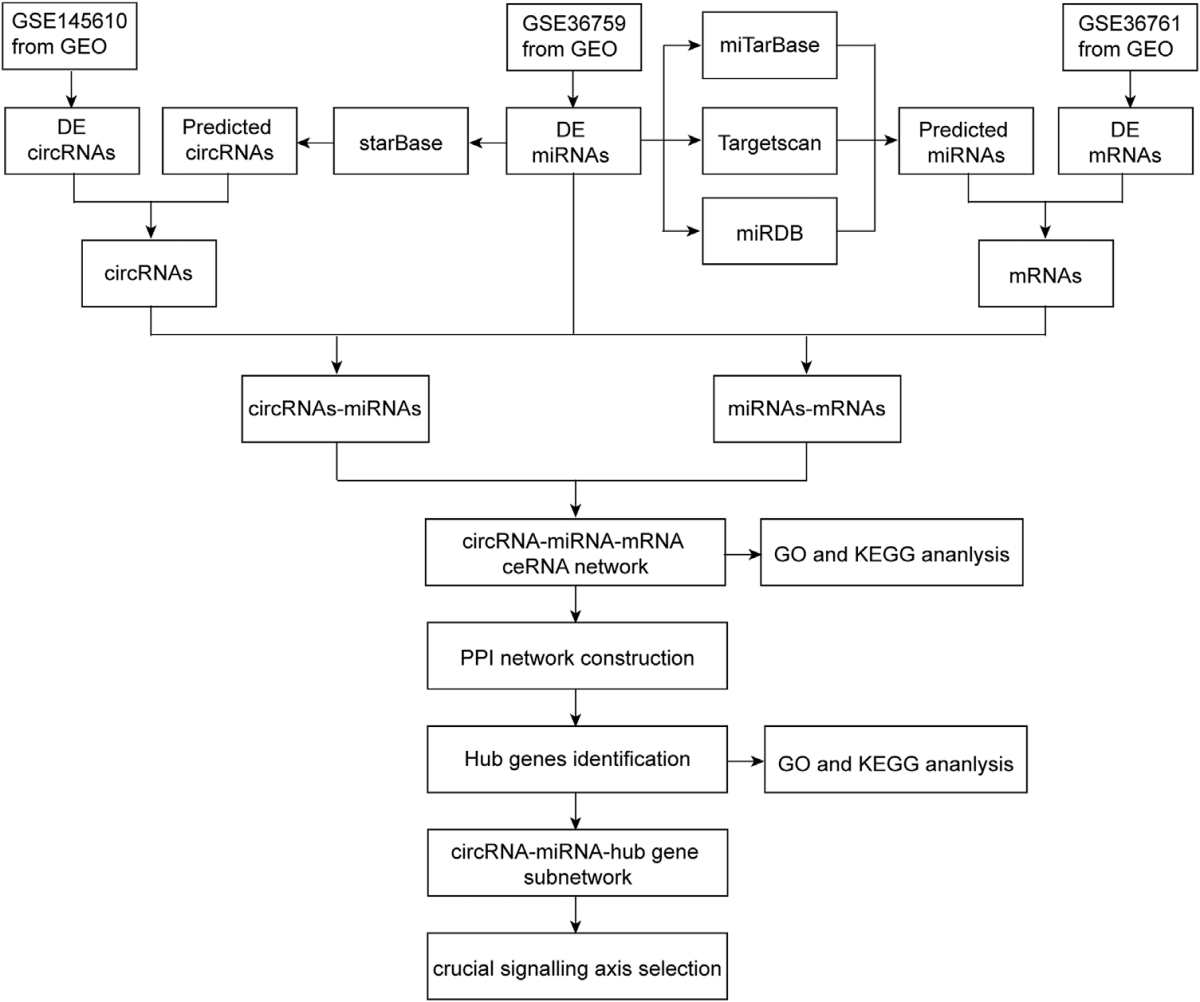
Flowchart of the present study to construct a circRNA-miRNA-mRNA regulatory network and identify crucial pathway axis of TOF.

The flow chart of this research process is shown in Figure 1.

#### 2.1.1 Collection of Datasets

The datasets used in this study were extracted from the GEO database according to the following criteria: 1) miRNA, mRNA, and circRNA expression profiles could be obtained by microarrays or RNA-sequencing (RNA-seq), and 2) healthy controls and patients with TOF in the datasets could be analyzed. Finally, one microarray dataset [GSE145610 (Yu et al., 2021)] and two RNA-seq datasets [GSE36759 (Grunert et al., 2019) and GSE36761 (Grunert et al., 2014)] were included in our study, in which GSE36759 was the miRNA expression profile. In this study, 66 samples (19 controls and 47 patients) were analyzed. The details of all included datasets are shown in Table 1.

**TABLE 1:** Microarray and RNA sequencing datasets from GEO were incorporated into this study.

	GEO accession number	Sample size		Platform
		Controls	Patients	
1	GSE36759	8	22	Illumina Genome Analyzer
2	GSE36761	8	22	Illumina Genome Analyzer
3	GSE145610	3	3	074301 Arraystar Human CircRNA microarray V2

GEO, gene expression omnibus dataset.

#### 2.1.2 Identification of Differentially Expressed circRNAs, miRNAs, and mRNAs

We used the following three tools to identify the differentially expressed RNAs in the selected datasets. BioJupies (http://biojupies.cloud), which was used to analyze miRNAs in GSE36759 dataset, is a freely available web-based application with 14 RNA-seq analysis library plug-ins that we used for the RNA-seq data analysis (Torre et al., 2018). NetworkAnalyst (https://www.networkanalyst.ca) (Zhou G. et al., 2019), which was used to analyze mRNAs in GSE36761, is a website with integrative statistical and visualization tools. Differential expression analysis of mRNAs or miRNAs between the TOF and control groups was conducted using the R package DESeq2. GEO2R (http://www.ncbi.nlm.nih.gov/geo/geo2r/) (Barrett et al., 2013) online tools were used to analyze circRNAs in GSE145610 dataset. Differential expression analysis of circRNAs between the TOF and control groups was conducted using the R/Limma package. The analyzed data was normalized in terms of log2FoldChange (logFC).

The adjusted *p*-value <0.05 and |logFC| > 1 were set as the cut-off criteria for screening differentially expressed mRNAs (DEmRNAs) and miRNAs (DEmiRNAs), while differentially expressed circRNAs (DEcircRNAs) were screened with thresholds of *p*-value <0.05 and |logFC| > 1.

#### 2.1.3 miRNA–Target and circRNA–miRNA Regulatory Network Analysis

The association of circRNA–miRNA–target genes was predicted using multiple online bioinformatic platforms. First, we extracted DEmiRNAs from the GSE36759 dataset. Three online websites [TargetScan (http://www.targetscan.org/) (Agarwal et al., 2015), miRTarBase (http://mirtarbase.mbc.nctu.edu.tw/) (Huang et al., 2020) and miRDB (http://www.mirdb.org/) (Chen and Wang, 2020)] were used to predict miRNA–mRNA interactions respectively. The predicted genes were selected from common miRNA–mRNA pairs in the three databases. Candidate mRNAs were identified by taking the interaction between predicted downstream genes and DEmRNAs from GSE36761. Second, circRNA–miRNA pairs were established using the starBase (http://starbase.sysu.edu.cn/) (Li J.-H. et al., 2014) online database. Candidate circRNAs were identified according to the intersection between predicted target circRNAs and DEcircRNAs from GSE145610. Finally, DEmiRNAs were combined with candidate mRNAs and circRNAs respectively to build miRNAs-mRNAs and circRNAs–miRNAs interaction pairs.

#### 2.1.4 Construction of the circRNA–miRNA–mRNA Regulatory Network

The circRNAs–miRNAs and miRNAs–mRNAs pairs were integrated to create the ceRNA (circRNA–miRNA–mRNA) regulatory network and visualized using Cytoscape software (Shannon et al., 2003).

#### 2.1.5 Gene Ontology and Kyoto Encyclopedia of Genes and Genomes Pathway Enrichment Analysis

A functional analysis of genes was conducted using a Gene Ontology (GO) (The Gene Ontology Consortium, 2019) analysis, which revealed the biological process (BP), cellular component (CC), and molecular function (MF) that the genes were involved in. The Kyoto Encyclopedia of Genes and Genomes (KEGG) (Kanehisa and Goto, 2000) pathway enrichment analysis revealed the signaling pathways in which the genes were involved. To further understand the functions of genes in TOF, mRNAs in the ceRNA network were analyzed by GO and KEGG enrichment analyses using Metascape (https://metascape.org) (Zhou Y. et al., 2019), with *p*-value < 0.05 as the cut-off criterion.

#### 2.1.6 Construction and Analysis of the Protein-Protein Interaction Network and Identification of Hub Genes

A PPI network of mRNAs in the ceRNA network was constructed using the STRING database (https://string-db.org/) (Szklarczyk et al., 2021) with the cut-off standard being a confidence score >0.4. The network was visualized using Cytoscape software. The cytoHubba plug-in in Cytoscape was used to measure the hub genes for mRNAs in the ceRNA network (Chin et al., 2014). Key molecules in the PPI network were detected using the Molecular Complex Detection (MCODE) application in Cytoscape (standard: degree cut-off = 2, max. depth = 100, k-core = 2, and node score cut-off = 0.2). Hub genes were obtained by analyzing the interactions between essential genes analyzed from cytoHubba and MCODE. In order to further explore the biological functions of hub genes, functional enrichment analysis was performed using the Metascape online tool.

### 2.2 Results

#### 2.2.1 Differentially Expressed circRNAs, miRNAs, and mRNAs in Tetralogy of Fallot

**FIGURE 2:**
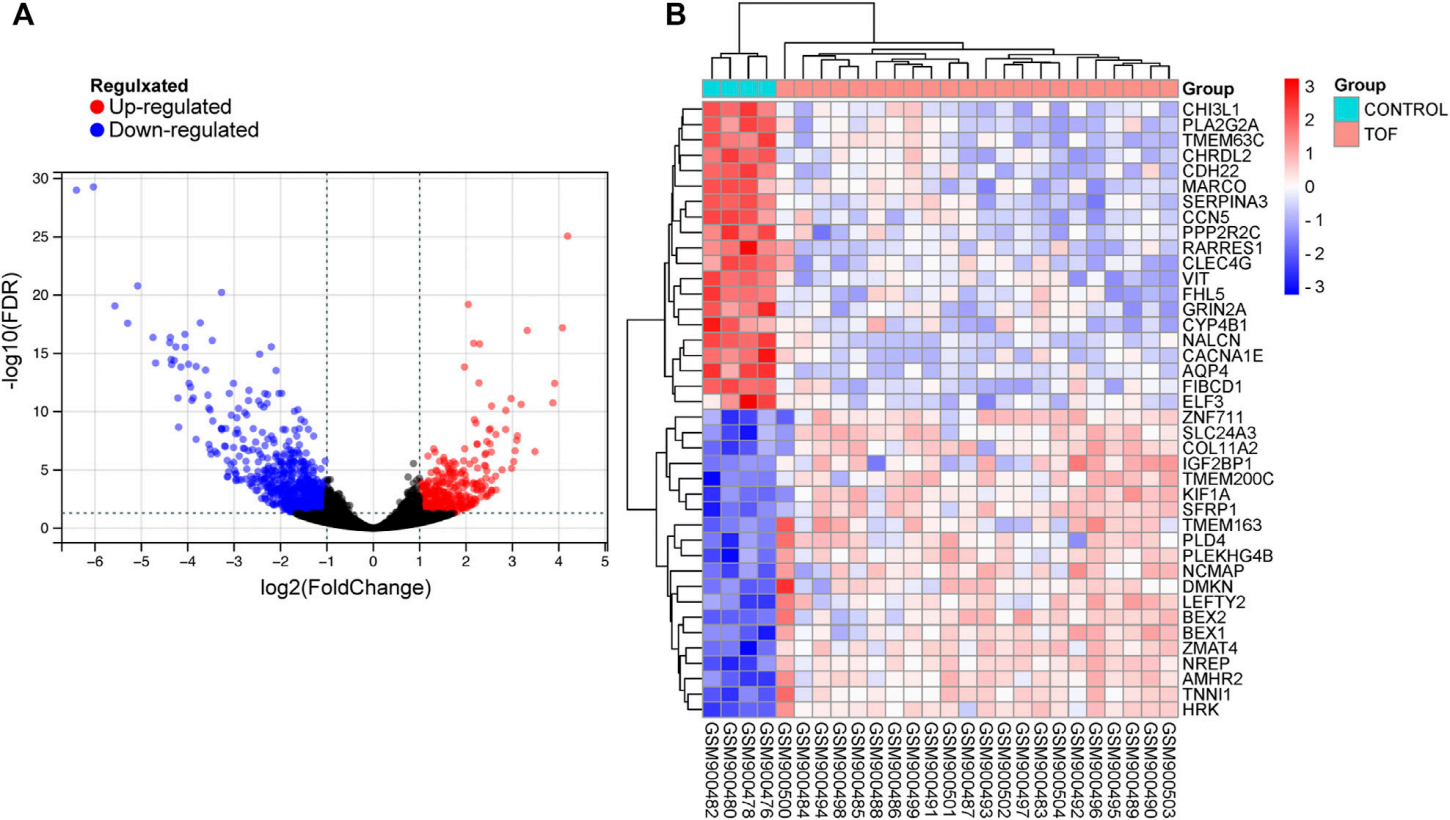
Acquire differentially expressed genes of TOF (DEmRNAs). **(A)** Volcano plots for DEmRNAs, the red and green points represent up and down expressed DEmRNAs respectively. **(B)** A heatmap for 40 DEmRNAs we selected, the change in color represents the difference in expression.

**FIGURE 3:**
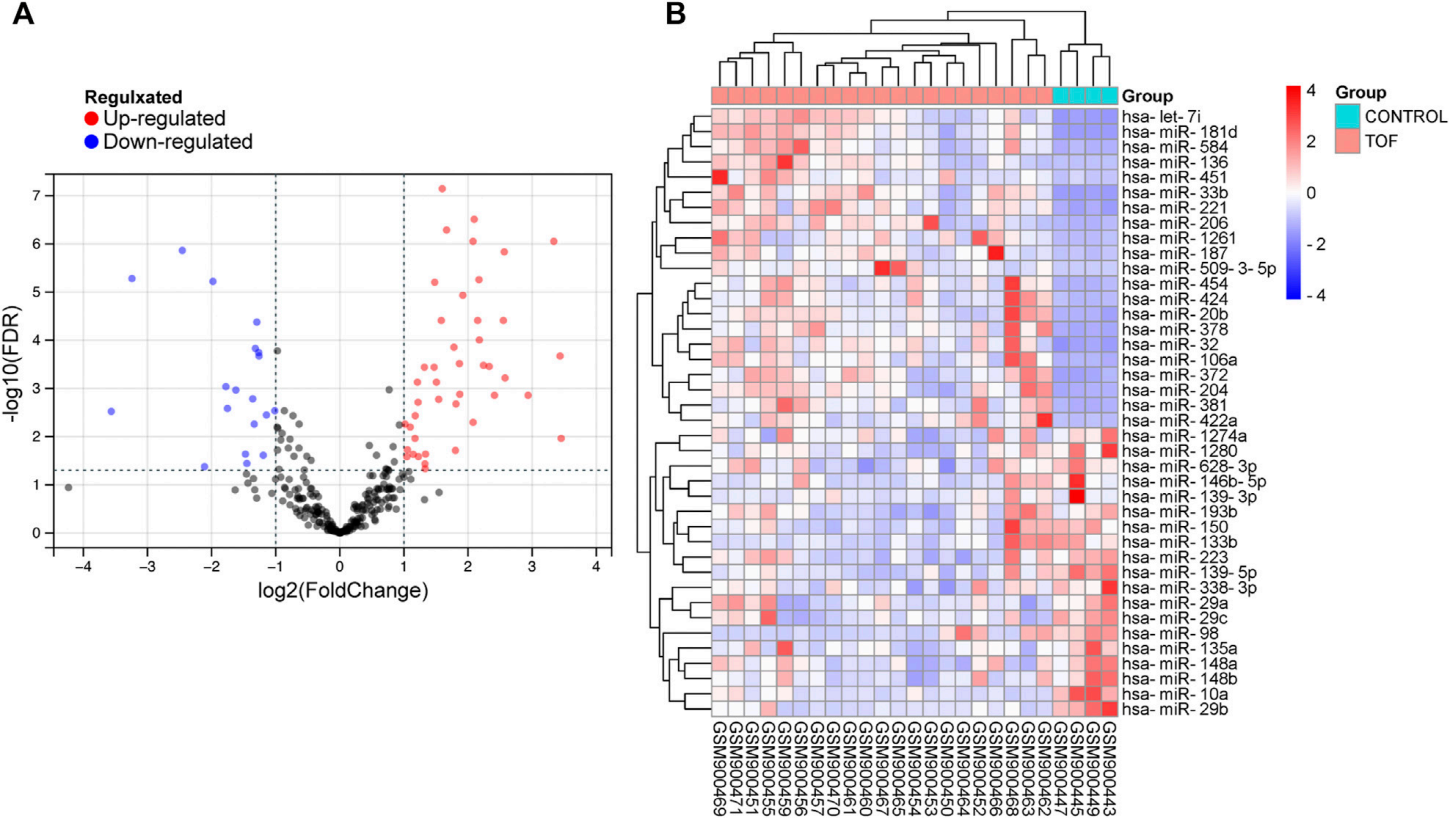
Acquire differentially expressed microRNAs (DEmiRNAs) of TOF. **(A)** Volcano plots for DEmiRNAs, the red and green points represent up and down expressed DEmiRNAs respectively. **(B)** A heatmap for 40 DEmiRNAs we selected, the change in color represents the difference in expression.

**FIGURE 4:**
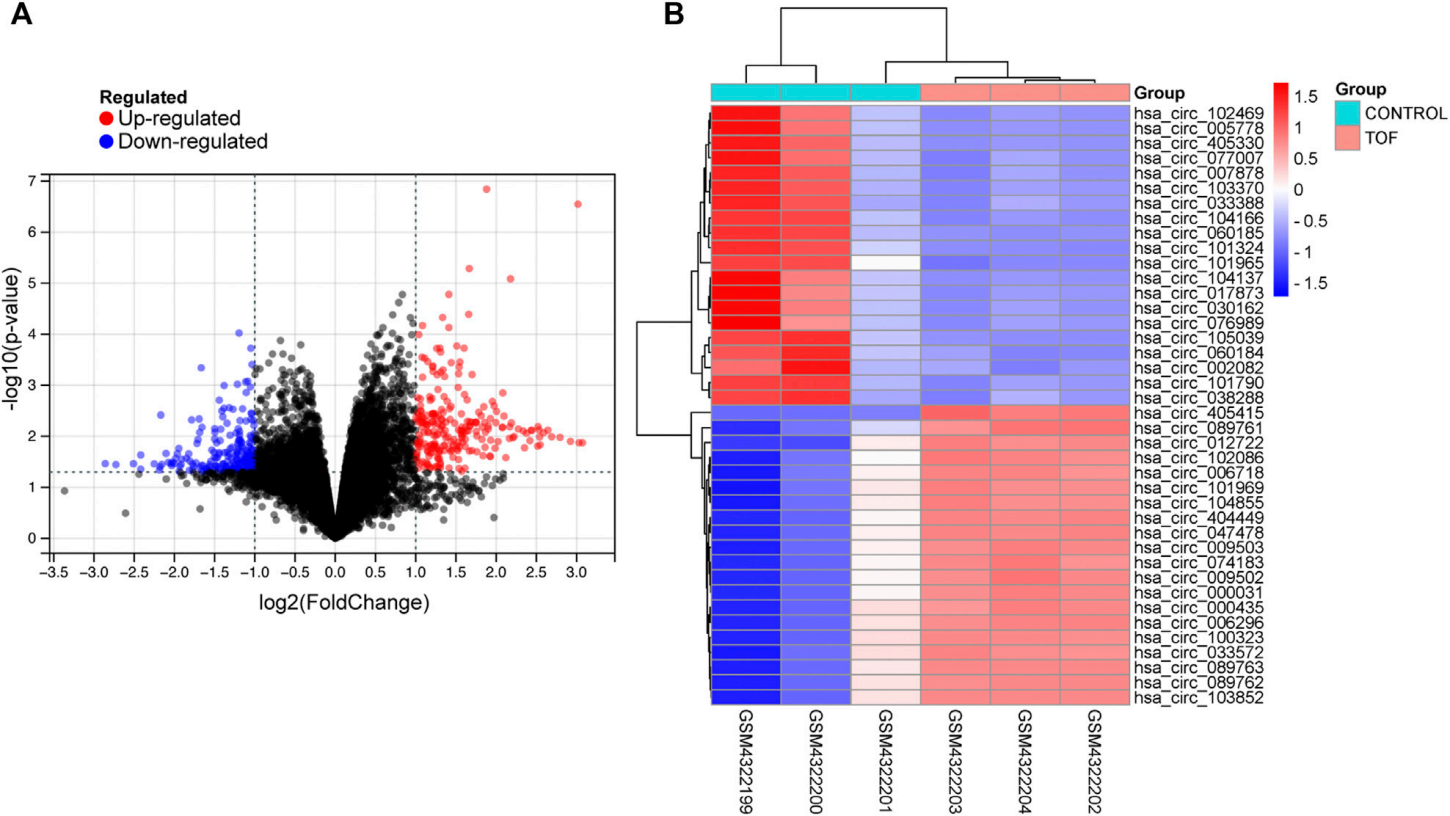
Acquire differentially expressed circRNAs (DEcircRNA) of TOF. **(A)** Volcano plots for DEcircRNAs, the red and green points represent up and down expressed DEcircRNAs respectively. **(B)** A heatmap for 40 DEcircRNAs we selected, the change in color represents the difference in expression.

After performing the quality control and data normalization, there were 1187 DEmRNAs, 411 upregulated genes, and 776 downregulated genes in the GSE36761 dataset compared to healthy heart tissues (Figure 2 and Supplementary Table S1). The most significantly upregulated and downregulated mRNAs were TNNI1 and AQP4. In the GSE36759 dataset, there were 63 DEmiRNAs, 44 upregulated genes, and 19 downregulated genes (Figure 3 and Supplementary Table S2). The most significantly upregulated and downregulated miRNAs were hsa-miR-1261 and hsa-miR-98. A total of 465 DEcircRNAs (244 upregulated and 221 downregulated circRNAs) in TOF were screened (Figure 4 and Supplementary Table S3), among which the most significantly upregulated and downregulated circRNAs were hsa_circ_089761 and hsa_circ_077007, respectively.

#### 2.2.2 Construction of the circRNA–miRNA–mRNA Regulatory Network in Tetralogy of Fallot

**FIGURE 5:**
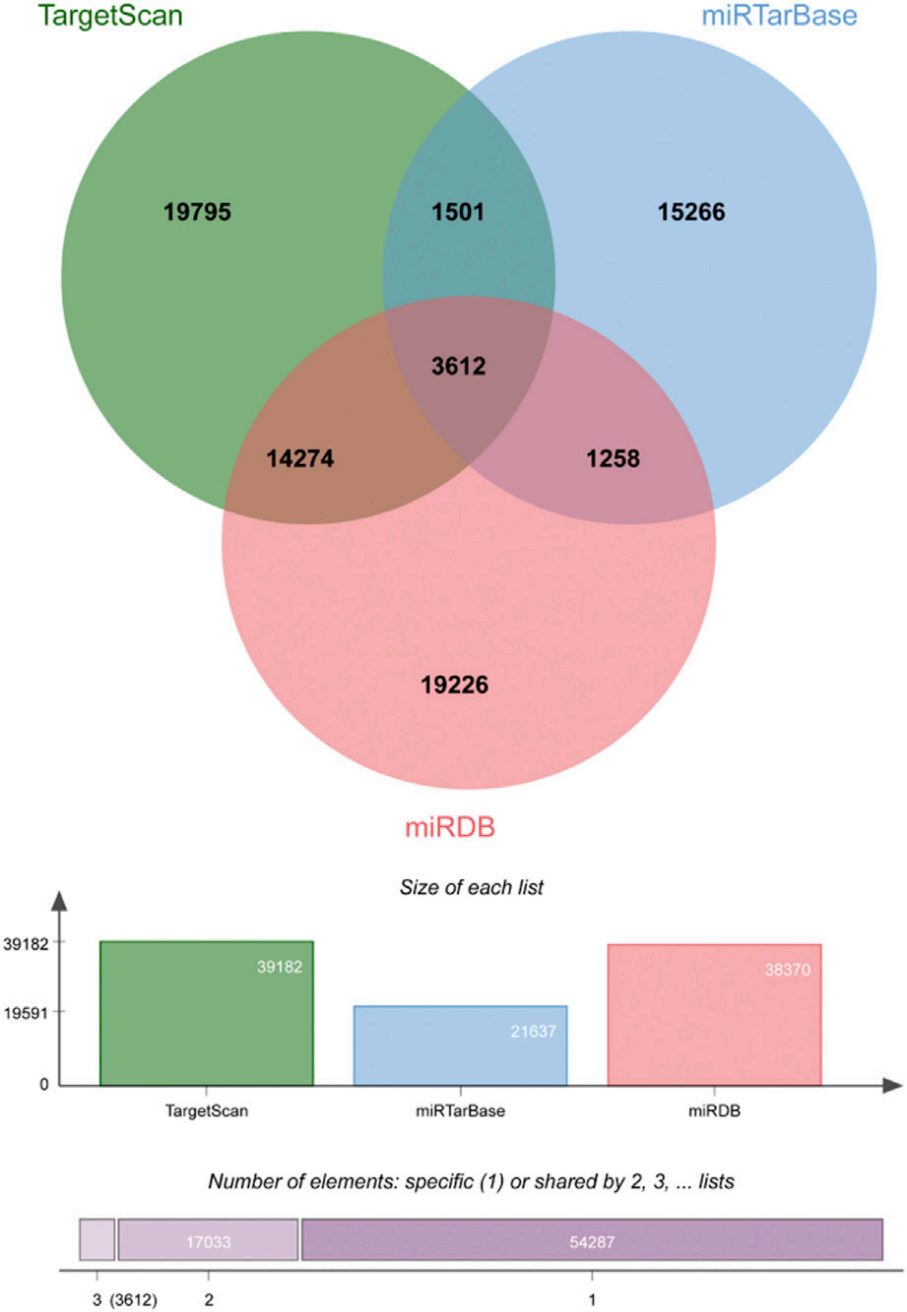
Veen plot of mRNAs predicted by DEmiRNAs.

**FIGURE 6:**
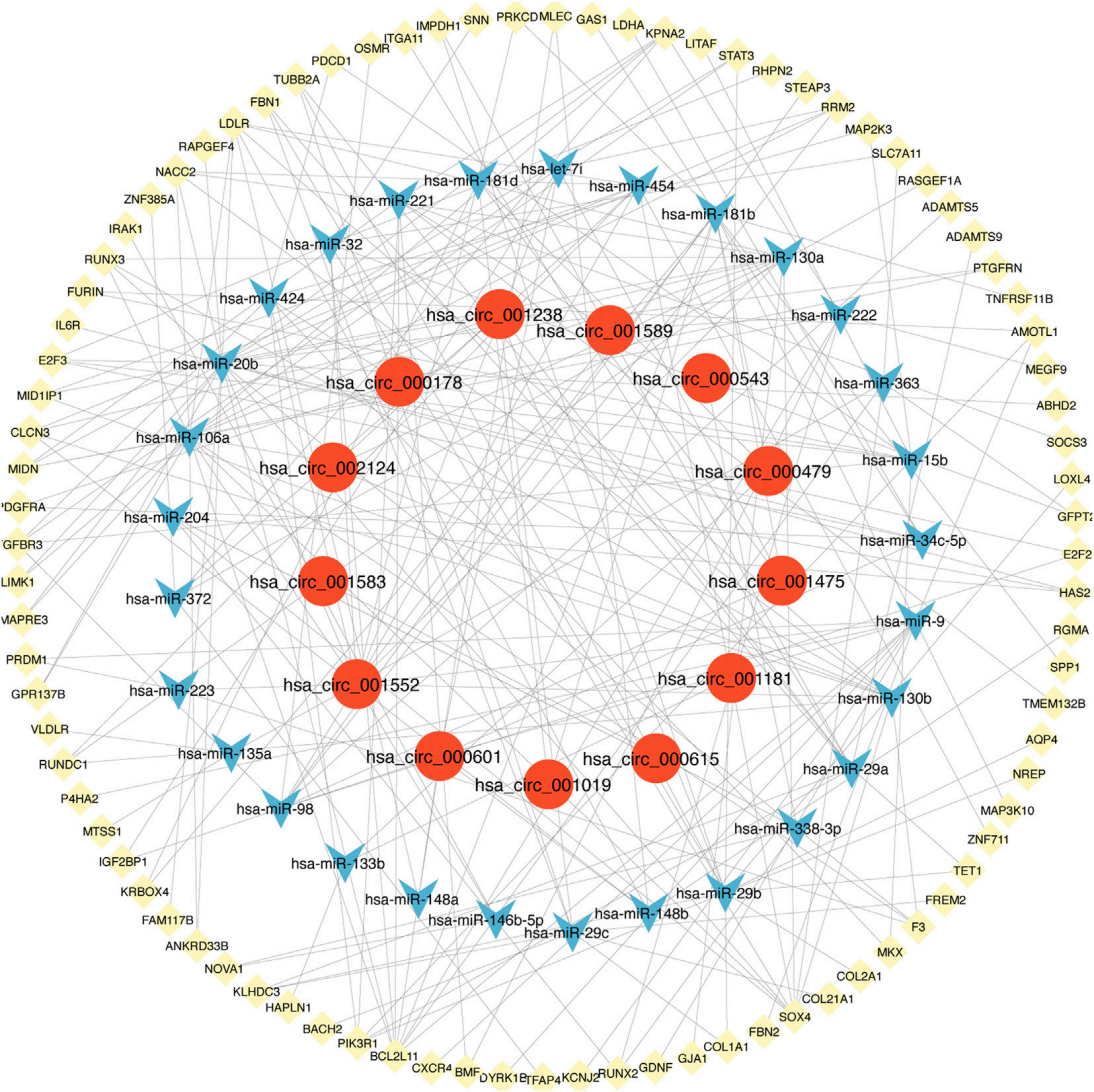
The view of DEcircRNA-DEmiRNA-DEmRNA regulatory network. The network includes 29 miRNAs, 13 circRNAs, 88 mRNAs and 231 edges. The yellow diamonds represented mRNA, the blue V shape represented miRNA, and the red circles represented circRNA.

There were 3612 common miRNA–mRNA pairs that were identified by the Venn diagram in the three prediction databases (Figure 5). A total of 97 candidate mRNAs were identified through these interactions. A total of 1339 circRNAs regulated by TOF-related miRNAs were predicted using the starBase database, and 16 candidate circRNAs were detected by intersection with DEcircRNAs obtained from GSE145610. We obtained 205 DEmiRNA–DEmRNA and 61 DEmiRNA–DEcircRNA interacting pairs, from which miRNAs paired with both circRNAs and mRNAs were extracted. A triple network of DEcircRNA–DEmiRNA–DEmRNA, including 29 miRNAs, 13 circRNAs, 88 mRNAs, and 231 interconnected pairs, was then constructed (Figure 6).

**FIGURE 7:**
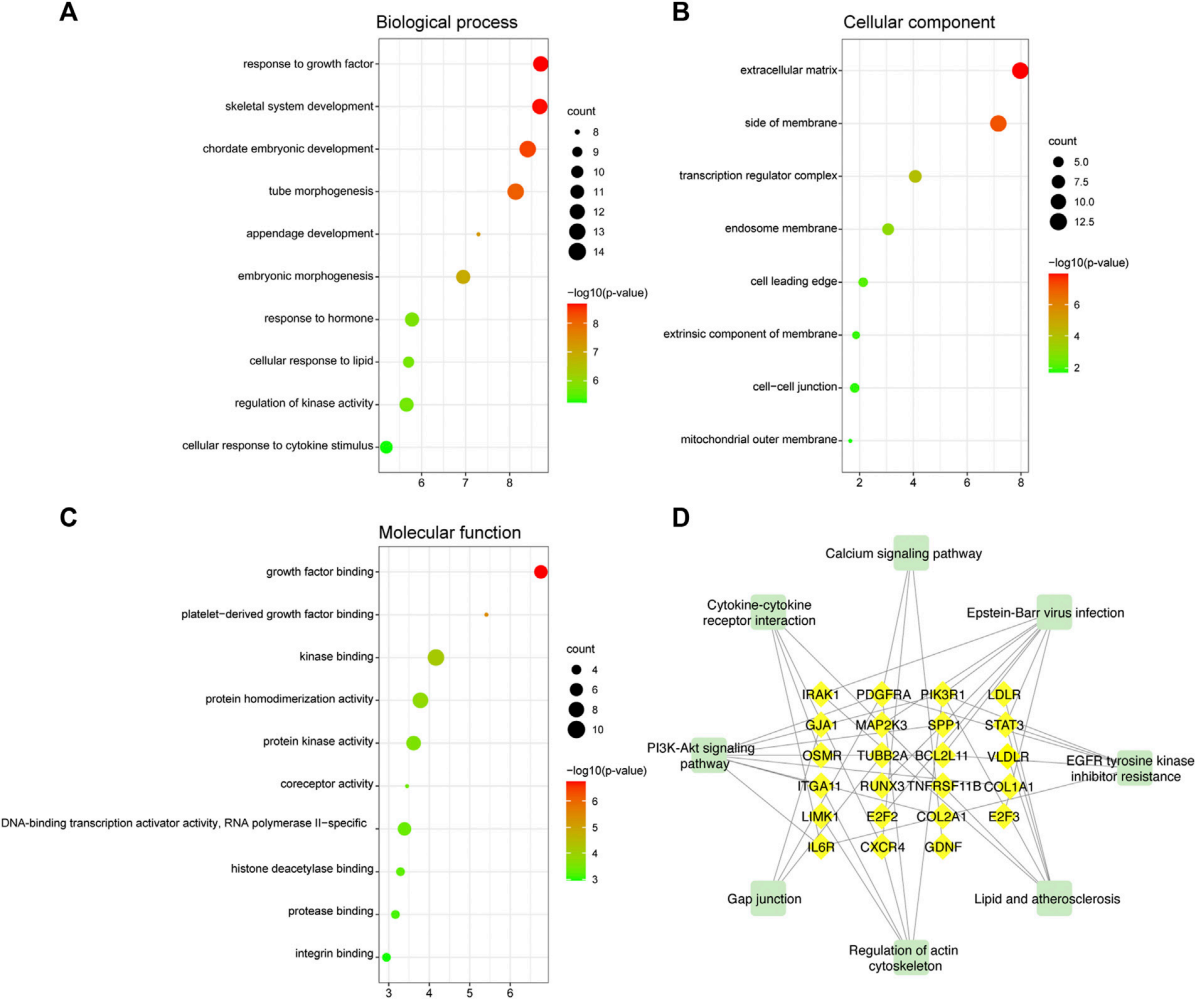
GO and KEGG functional enrichment analyses of mRNAs in the ceRNA regulatory network. **(A)** Biological process analysis. **(B)** Cellular component analysis. **(C)** Molecular function analysis. The color intensity of the nodes shows how rich the analysis is. The enrichment factor is defined as the ratio of differential genes in the whole genome. The size of dot represents the number of genes in the pathway. **(D)** KEGG pathway analysis. The green rectangle represented the pathway and the yellow diamonds represented mRNA.

We explored the pathogenesis of TOF through GO and KEGG enrichment analyses of the 88 target genes. The main enriched BP categories were response to growth factor, skeletal system development, and chordate embryonic development. The CC items mainly included the extracellular matrix, side of membrane, and transcription regulator complex. The MF analysis mainly revealed that the genes were involved in growth factor binding, platelet-derived growth factor binding, and kinase binding. The most enriched KEGG pathways included Epstein-Barr virus infection, PI3K-Akt signaling pathway, EGFR tyrosine kinase inhibitor resistance, Lipid and atherosclerosis, and regulation of actin cytoskeleton. The significantly enriched terms in the GO and KEGG enrichment analysis are shown in Figure 7 (Supplementary Table S4).

#### 2.2.3 Enrichment Analysis of the Protein-Protein Interaction Network and Identification of Hub Genes

**FIGURE 8:**
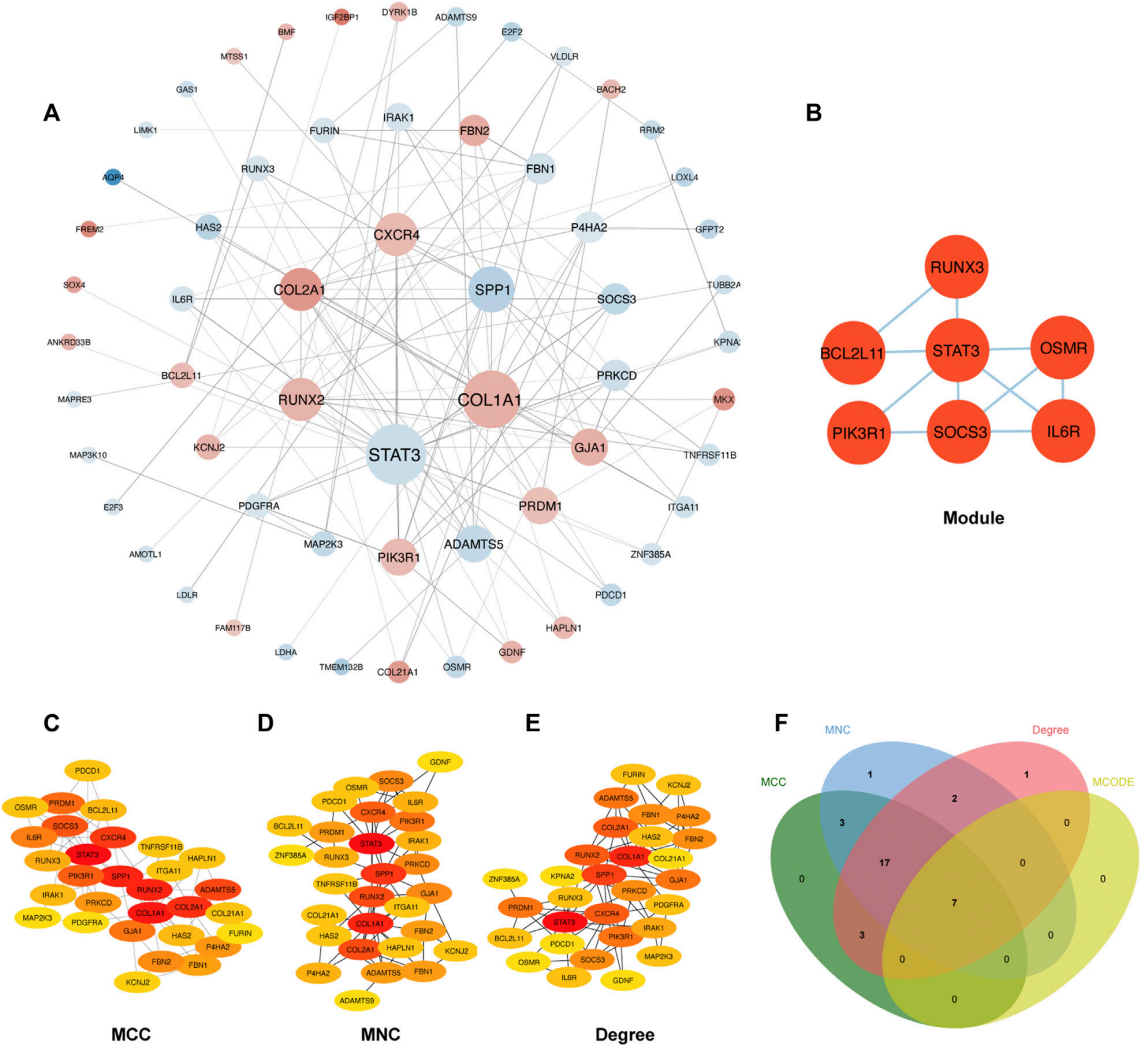
Construction of PPI network and identification of hub genes. **(A)** The PPI network of 88 mRNAs was selected by crossover. Red indicates upregulation, blue indicates downregulation, and color depth indicates upregulation degree. The size of the circle indicates the size of the degree value. **(B)** Key module of the PPI network. **(C–E)** The hub genes were identified using four models (Degree, MCC, MNC, and Degree). **(F)** Venn diagram was used to identify the 7 hub genes in TOF.

The PPI network involving 88 mRNAs in the ceRNA regulatory network was constructed using the STRING database and imported into Cytoscape software for visualization (Figure 8A). The PPI enrichment *p*-value was <1.0 × 10^−16^, indicating that these proteins were at least partially bioconjugated as a group, and that the interactions between the proteins themselves were greater than those with random proteins. In total, 60 nodes and 118 PPI relationships were identified. Owing to the existence of biological networks, it is necessary to use different topological analysis methods to identify hub genes. We identified a key module containing 7 genes and 11 edges using the MCODE application in Cytoscape (Figure 8B). There are 11 types of topological analysis methods in the cytoHubba plug-in of the Cytoscape software. In this study, the top 30 genes were obtained using three relatively accurate topological analysis methods, including MCC, MNC, and degree, to construct the subnetworks of the PPI network (Figures 8C–E). Seven hub genes that may be related to TOF were explored by the intersection of one key module and the top 30 genes analyzed using three different methods (Figure 8F). These genes included STAT3, SOCS3, PIK3R1, IL6R, RUNX3, OSMR, and BCL2L11. Of these seven genes, two were upregulated (BCL2L11and PIK3R1) and five were downregulated (STAT3, SOCS3, L6R, RUNX3, OSMR, and BCL2L11) (Table 2 and Supplementary Table S5).

**TABLE 2:** Hub genes identified from the PPI network analysis.

Gene	Degree	LogFC	adj *p*-Value	Regulation
BCL2L11	4	1.2482	0.000016839	Up
PIK3R1	8	1.3103	0.000084773	Up
SOCS3	6	−1.9495	0.0055589	Down
OSMR	3	−1.8603	0.000026841	Down
STAT3	16	−1.5595	1.9723E-06	Down
RUNX3	4	−1.2083	0.016322	Down
IL6R	4	−1.1787	0.009665	Down

adj *p*-value, adjusted *p*-value; log FC, log (Fold Change).

**FIGURE 9:**
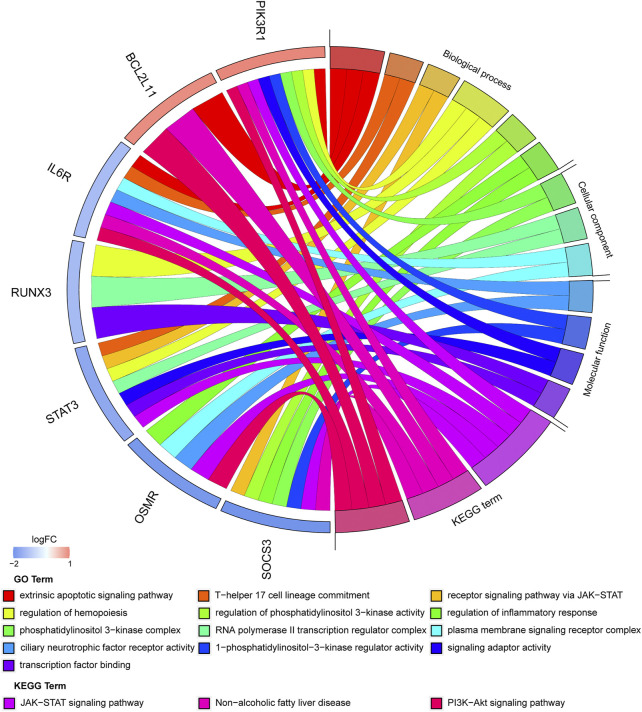
Significantly enriched items GO analysis and KEGG pathway analysis of hub genes.

An enrichment analysis of the above seven hub genes was performed using the online Metascape tool. Regarding GO terms, the mainly enriched ones were extrinsic apoptotic signaling pathway, T-helper 17 cell lineage commitment, ciliary neurotrophic factor receptor activity, 1-phosphatidylinositol-3-kinase regulator activity, and phosphatidylinositol 3-kinase complex. The KEGG signaling pathway analysis showed that the JAK-STAT signaling pathway and PI3K-Akt signaling pathway were significantly enriched. The results are presented in Figure 9 (Supplementary Table S6). We propose that these hub genes may play vital roles in the pathogenesis of TOF and therefore warrant further investigation.

#### 2.2.4 Construction of the circRNA–miRNA–Hub Gene Subnetwork and Identification of the Crucial Signaling Pathway Axis

**FIGURE 10:**
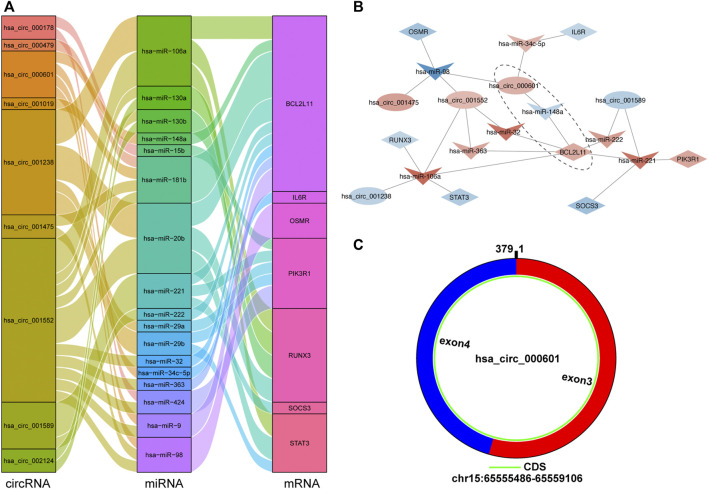
The circRNA-miRNA-hub gene subnetwork and crucial signaling pathway axis. **(A)** The Sankey diagram of the subnetwork in TOF. Each rectangle represents an element (circRNA, miRNA, mRNA), and the size of the rectangle indicates the degree of connection of each component. **(B)** Identification of crucial signaling pathway axis. Red indicates up-regulation, blue indicates down-regulation, and color depth indicates up-regulation degree. **(C)** Predicted the structure of hsa_circ_000601 based on the CircPrimer software.

A circRNA–miRNA–hub gene subnetwork was constructed with 9 circRNAs, 17 miRNAs, and 7 mRNAs (Figure 10A). According to ceRNA theory, circRNAs mainly regulate miRNAs expression by acting as sponges. Therefore, we recognized the ceRNA regulatory pairs hsa_circ_000601/hsa-miR-148a/BCL2L11 in the subnetwork as crucial signaling pathway axis (Figure 10B). After submitting a query in circBase (http://www.circbase.org), it was shown that hsa_circ_000601 is located on chromosome 15, consists of two exons, and contains a complete 379-bp sequence (Figure 10C).

### 2.3 Discussion

As we all know, cardiac development is a complex and dynamic process. Challenges in the field of cardiology remain given the lack of effective biomarkers. In the case of TOF, its underlying molecular mechanisms remain unclear. Prenatal diagnoses and genetic counseling for TOF have always been difficult; therefore, it is important to explore its specific molecular mechanisms. A number of genetic and genomic factors are involved in cardiac development, including mutations, histone modifications, DNA/RNA methylation, ncRNA modifications and so on (O'Brien et al., 2012; Gao J. et al., 2017; Zaidi and Brueckner, 2017). In CHD, ncRNAs may be potential biomarkers for better diagnoses and prognoses. The expression profiles of mRNAs, miRNAs, and circRNAs have also been explored in TOF (Grunert et al., 2016; Liu et al., 2018; Grunert et al., 2019). It is worth noting that the ceRNA regulatory network plays an important role in TOF development.

In our study, a comprehensive analysis of microarray and RNA-seq datasets was performed to determine the regulatory network of TOF. We constructed a TOF regulatory network consisting of 29 miRNAs, 13 circRNAs, and 88 mRNAs. Then, the function of the ceRNA network was further predicted by GO and KEGG enrichment analyses of the target genes. In addition, seven hub genes were obtained from PPI networks using the cytoHubba and MCODE plug-ins of Cytoscape. These hub genes were mainly involved in the regulation of phosphatidylinositol 3-kinase activity (in BP terms), 1-phosphatidylinositol-3-kinase regulator activity (in MF terms) and phosphatidylinositol 3-kinase complex (in CC terms). Phosphatidylinositol 3-kinase (PI3K) promotes cell growth by coupling macromolecular biosynthesis with the initiation of the cell cycle and is essential for translating extracellular growth signals into intracellular actions (Goncalves et al., 2018). The results of the GO functional analysis also indicated that two hub genes (STAT3and SOCS3) were significantly enriched in the receptor signaling pathway *via* JAK-STAT. The results of the GO and KEGG pathway further showed that six hub genes (IL6R, PIK3R1, STAT3, SOCS3, OSMR, and BCL2L11) were significantly enriched in apoptotic-related pathways, including extrinsic apoptotic signaling pathway, JAK-STAT signaling pathway and PI3K-Akt signaling pathway. Both JAK-STAT3 signaling and PI3K-Akt signaling pathways are involved in the regulation of hypoxia and apoptosis. The activation of two signaling pathways have been shown to attenuate hypoxia-induced apoptosis (Ke et al., 2019; Li et al., 2021).

BCL2L11 (Bim), a member of the pro-apoptotic Bcl-2 family, is located in the outer mitochondrial membrane. This protein mediates excitotoxic apoptosis by acting as a central regulator of the internal apoptotic cascade (Li Y. et al., 2014). Various studies have shown that BCL211 is closely related to hypoxia-induced myocardial apoptosis (Meng et al., 2020; Huang et al., 2021). PIK3R1 (also called PI3K p85α) is a regulatory subunit of the PI3K-Akt signaling pathway (Fan et al., 2020) that has activating and inhibitory effects on tumors (Vallejo-Díaz et al., 2019). PIK3R1 is one of the upstream regulators of the PI3K-Akt pathway, and its mutation may lead to the dysregulation of the PI3K pathway, thereby affecting apoptosis (Shull et al., 2012). STAT3 plays an important role in cardioprotection and is activated by phosphorylation as a downstream consequence of the JAK-STAT signaling pathway (Ou et al., 2021). As a transcription factor, STAT3 upregulates pro-angiogenic, anti-oxidant and anti-apoptotic genes, but suppresses anti-fibrotic and anti-inflammatory genes (Harhous et al., 2019). IL6R inhibits apoptosis by mediating IL-6-dependent STAT3 activation (Zhu et al., 2017). Recent evidence has suggested that apoptosis is associated with the inhibition of the IL-6/IL-6R autocrine signaling axis (Bharti et al., 2016). OSM is the produced by some tumor cells and inflammatory cells and belongs to the IL-6 cytokine family (Jones and Jenkins, 2018). OSMR is the receptor for OSM. Both JAK and STAT3 can be phosphorylated by the complex of IL-6 with its receptor IL-6R and glycoprotein 130 (Kaur et al., 2020), OSM with OSMR and gp130 (Rose-John, 2018). A major negative regulator of STAT3 during tumorigenesis is the inhibitor of cytokine signaling 3 (SOCS3) (Wan et al., 2015). Previous studies have shown that activation of the STAT3 cascade can be activated by reducing SOCS3 during hypoxia (Yokogami et al., 2013).

The hub genes screened in this study are involved in the regulation of hypoxia and apoptosis. Impairments in oxygen delivery in patients with CHD are a consequence of the abnormal anatomy of the heart. In our analysis, apoptosis can cause malformation of heart development, abnormal cardiac anatomy in patients with CHD can lead to oxygen delivery disorder. In addition, hypoxia can induce apoptosis of cells in related pathways, so TOF should be the result of interaction between apoptosis and hypoxia. TOF is a congenital heart malformation characterized by chronic hypoxia. A large number of research reports have pointed out that in cyanotic children with TOF, apoptosis is induced by chronic hypoxia, and the expression of related genes is remodeled (Ghorbel et al., 2010). Hypoxia-inducible factor-1α (HIF-1α) is also closely linked to TOF (Vaikom House et al., 2022). We further investigated the relationship between these hub genes and classical signaling pathways associated with hypoxia, such as HIF-1 signaling. Three hub genes (PIK3R1, IL-6R, and STAT3) were upstream regulators of the HIF-1 pathway. Studies have shown that can also regulate the expression of HIF-1α (Gao X. et al., 2017; Zhang et al., 2018; Grillo et al., 2020). The severity of hypoxia affects the occurrence of myocardial apoptosis (Tsang et al., 2014). Hypoxia increases cardiomyocyte apoptosis (He et al., 2013), resulting in myocardial cell loss. Inhibiting apoptosis can decrease myocardial damage and alleviate cardiomyocyte removal caused by hypoxic injury (Kanazawa et al., 2017). Therefore, the inhibition of hypoxia-induced apoptosis in cardiomyocytes may be useful for treating CHD.

To date, the molecular characteristics of circRNA-associated ceRNA networks in TOF have been poorly studied. A circRNA–miRNA–mRNA regulatory network in TOF was constructed in this study. A circRNA–miRNA–hub gene subnetwork was further constructed with 9 circRNAs, 17 miRNAs, and 7 mRNAs. It was then identified as a crucial signaling pathway axis. Several studies have found that miRNAs are central regulators that play vital roles in CHD formation in many cardiac developmental processes. A total of 17 DEmiRNAs were identified in the ceRNA subnetwork, of which 13 were upregulated and 4 were downregulated in the TOF samples. Among them, it has been confirmed that hsa-miR-98, hsa-miR-29b, hsa-miR-148a, hsa-miR-9, hsa-miR-222, hsa-miR-130a, hsa-miR-181b, hsa-miR-424, and hsa-miR-20b are related to heart function and developmental processes. Hsa-miR-98 is considered an important member of the tumor family and protects cardiomyocytes from apoptosis by regulating the expression of anti-apoptotic Bcl-2, pro-apoptotic Bax proteins, and the Fas/caspase-3 apoptotic signaling pathway (Sun et al., 2017). Hsa-miR-222 and hsa-miR-424 are upregulated in the ventricular outflow tract tissue of infants with TOF, and *in vitro* experiments have shown that hsa-miR-222 and hsa-miR-424 are involved in cardiomyocyte proliferation, differentiation, and migration (Zhang et al., 2013). It was reported that hsa-miR-29b and its target genes NOTCH2, Col1A2, and IGF1 can further regulate the proliferation (Yang et al., 2020) and cardiac fibrosis (Altaf et al., 2019) of cardiomyocytes. Hsa-miR-148a has been identified as a possible biomarker of ventricular septal defects (VSD) (Radhakrishna et al., 2019). Hsa-miR-9 inhibits the hypoxia-induced apoptosis of cardiomyocytes by targeting YAP1, and its expression is increased in the cardiac tissues of patients with CHD (Zheng et al., 2019). By activating the BMP signaling pathway, hsa-miR-20b overexpression promotes cardiomyocyte apoptosis and differentiation (Zhu et al., 2015). Similarly, hsa-miR-181b has been found to correlate with hypoxia-triggered cardiomyocyte apoptosis (Lv et al., 2020). Hsa-miR-130a affects cardiac sinus venous and inflow tract differentiation (Garcia-Padilla et al., 2021). In summary, we speculate that these miRNAs are mainly involved in the division, differentiation, and apoptosis of cardiomyocytes during the pathogenesis of TOF.

CircRNAs are frequently used as biomarkers for diagnoses and prognoses owing to their diversity, tissue-specific expression, and stability of their covalently closed-loop structures (Jiang et al., 2018). In this study, nine DEcircRNAs (hsa_circ_001552, hsa_circ_001238, hsa_circ_002124, hsa_circ_000601, hsa_circ_000178, hsa_circ_000479, hsa_circ_001019, hsa_circ_001475, and hsa_circ_001589) were identified. To the best of our knowledge, these circRNAs are the first reported abnormal factors found in TOF and have not yet been studied, making them potential novel biomarkers or therapeutic targets for TOF. Studies have shown that circRNAs act as miRNA sponges to inhibit the binding of miRNAs to target genes and regulate their expression (Xiong et al., 2018). Therefore, we believe that the circRNA–miRNA–mRNA axis may be a potential molecular regulatory mechanism of TOF. In this study, we identified one circRNA–miRNA–mRNA axis (hsa_circ_000601/hsa-miR-148a/BCL2L11) that may play an essential role in TOF. BCL2L11 and hsa-miR-148a are associated with cardiomyocyte apoptosis are. BCL2L11 is a potential target of hsa-miR-148a. In our study, BCL2L11 and hsa_circ_000601 were upregulated and hsa-miR-148a was downregulated. The downregulation of hsa-miR-148a can lead to decreased Bcl-2 levels and increased the expression of BCL2L11, thereby inducing apoptosis (Eichelmann et al., 2018). We suggest that hsa_circ_000601 may regulate the expression of these genes during hypoxia, and the upregulation of this circRNA may lead to the upregulation of BCL2L11 in TOF by reducing the intracellular level of hsa-miR-148a. A previous study also constructed a circRNA-associated ceRNA network and proposed that the hsa_circ_0007798/miR-199b-5p/HIF1A signaling pathway axis is related to hypoxia and cardiac defects (Yu et al., 2021). This complements our study and is conducive to further explorations of the molecular mechanisms of hypoxia and apoptosis in TOF.

### 2.4 Limitations

Our current study still has certain limitations. For example, the relatively small number of TOF specimens from patients may have reduced the confidence in the analysis of the ceRNA regulatory network. In addition, this study lacks further experimental results to providea solid foundation for the study. The sponge effect and regulatory function of specific circRNAs such as hsa_circ_000601, require further verification.

### 2.5 Conclusion

We comprehensively analyzed two microarray datasets and one RNA-seq dataset to identify specific circRNAs, miRNAs, and mRNAs that may be key nodes of TOF. Then we established a circRNA–miRNA–mRNA regulatory network and screened seven hub genes in TOF. In addition, we propose that the hsa_circ_000601/hsa-miR-148a/BCL2L11 signaling pathway axis may play a crucial role in regulating TOF progression. Overall, the ceRNA network and key regulatory pathways identified in our study may provide potential biomarkers and new insights for the early diagnosis and targeted therapy of TOF, which needs to be further explored in the future.

## Data Availability

The original contributions presented in the study are included in the article/Supplementary Material, further inquiries can be directed to the corresponding author.
